# Development, Analytical, and Clinical Evaluation of Rapid Immunochromatographic Antigen Test for SARS-CoV-2 Variants Detection

**DOI:** 10.3390/diagnostics12020381

**Published:** 2022-02-02

**Authors:** Jidapa Szekely, Jenureeyah Mongkolprasert, Nitikorn Jeayodae, Chadarat Senorit, Panuttha Chaimuti, Piyawut Swangphon, Natthaphon Nanakorn, Teerapat Nualnoi, Paweena Wongwitwichot, Theerakamol Pengsakul

**Affiliations:** 1 Faculty of Medical Technology, Prince of Songkla University, Hat Yai, Songkhla 90110, Thailand; piyawut.s@psu.ac.th (P.S.); natthaphon.s@psu.ac.th (N.N.); 2 Kestrel Bio Sciences Thailand Co., Ltd., Klong Luang, Pathumthani 12120, Thailand; jeh.kbsthailand@gmail.com (J.M.); korn.kbsthailand@gmail.com (N.J.); chadarat.kbsthailand@gmail.com (C.S.); 3 Immunology and Virology Unit, Department of Medical Technology and Clinical Pathology, Hat Yai Hospital, Hat Yai, Songkhla 90110, Thailand; syberiabox@gmail.com; 4 Department of Pharmaceutical Technology, Faculty of Pharmaceutical Sciences, Prince of Songkla University, Hat Yai, Songkhla 90110, Thailand; teerapat.n@psu.ac.th; 5 Department of Pharmaceutical Chemistry, Faculty of Pharmaceutical Sciences, Prince of Songkla University, Hatyai, Songkhla 90110, Thailand; paweena.w@psu.ac.th

**Keywords:** SARS-CoV-2, antigen test kit, antigen rapid diagnostic test, variant detection, nucleocapsid

## Abstract

The antigen rapid diagnostic test (Ag-RDT) is a useful diagnostic tool for the detection and management of COVID-19 spread. Global SARS-CoV-2 variant outbreaks have highlighted the need for a test capable of detecting SARS-CoV-2 variants with high sensitivity and a low limit of detection. This study aimed to develop and evaluate, both analytically and clinically, an antigen rapid diagnostic test (the Kestrel^TM^ COVID-19 Ag Rapid Test) for professional use. A lateral flow immunoassay-based diagnostic test kit was developed, and various aspects of its analytical performance were evaluated. This test kit was clinically evaluated by two independent laboratories and showed closely related results of 96.49% and 98.33% of sensitivity, 100% and 100% of specificity, and 99.01% and 99.44% of accuracy, respectively. A limit of detection was observed at values as low as 0.156 ng/mL for recombinant SARS-CoV-2 nucleocapsid protein. Moreover, the test kit successfully detected the recombinant SARS-CoV-2 nucleocapsid protein (NP) of wild-type, Alpha-, Beta-, Gamma-, Delta-, Epsilon-, Kappa-, and Omicron-variants as positive results. Therefore, the Kestrel^TM^ COVID-19 Ag Rapid Test may have potential use for effective COVID-19 screening, surveillance, and infection control in a variety of global SARS-CoV-2 variant outbreaks.

## 1. Introduction

A global pandemic of severe acute respiratory syndrome coronavirus 2 (SARS)-CoV-2 has had an enormous impact on public health and the economy worldwide [1]. The most challenging aspect of this pandemic in 2021—a trend that may continue to 2022—is dealing with the emergence of multiple variants of concern (VOCs) [2]. Importantly, a variant that shows partial immune escape could provoke a wave of infection [3]. The situation has led to an urgent need to detect early infection and for the detection method to be able to detect infection by variants of concern. A COVID-19 detection method that is affordable, reliable, rapid, and allows for the early detection of infection by variants of concern, is crucial to control and manage the COVID-19 pandemic.

The antigen rapid diagnostic test (Ag-RDT) is a useful diagnostic tool that can help detect and manage COVID-19 spread in terms of short turnaround times and favorable performance in detecting SARS-CoV-2 VOCs [4,5]. Although numerous brands of Ag-RDTs are available worldwide, reliable and research-supported Ag-RDTs are needed [6,7]. The protein target of the Ag-RDTs is a critical feature of detecting SARS-CoV-2 infection by variants of concern. Since mutations mostly occur in the spike protein of the virus, other structure proteins, particularly the nucleocapsid (N) protein, serve as better targets of detection due to the lower mutation rate. The newly detected Omicron variant contains only two mutations in the region coding for the N protein compared to more than 30 mutations in the spike protein, as reported at the time of the current publication [8].

Various aspects of the performance of Ag-RDTs for SARS-CoV-2, including sensitivity, specificity, and positive and negative predictive values, are important for selecting the most appropriate test. Although the sensitivity of Ag-RDTs is normally lower than that of laboratory-based Nucleic Acid Amplification Tests (NAATs), the specificity of the test is generally as high as that of NAATs. While Ag-RDTs are less sensitive than NAATs, particularly in asymptomatic populations, the WHO’s updated interim guidance recommends the use of Ag-RDTs that meet the minimum performance requirements of ≥80% sensitivity and ≥97% specificity in the diagnosis of SARS-CoV-2 infection [9]. Ag-RDTs meeting the minimum performance requirements can be used for primary case detection, contact tracing, outbreak investigations, and monitoring trends of disease incidence in communities.

Several lateral flow immunoassay-based diagnostic tests that detect antigens specific for SARS-CoV-2 infection have become commercially available. Generally, the Ag-RDTs for a clinical COVID-19 diagnosis are less sensitive than the viral NAATs. The sensitivity of the commercial Ag-RDTs compared to RT-PCR ranges from 42 to 90%, and their specificity ranges from 61.2 to 100% [10,11,12,13,14]. However, repeated VOC outbreaks highlight the need for a test capable of detecting SARS-CoV-2 VOC with high sensitivity and a low limit of detection. This study aimed to develop and evaluate, both analytically and clinically, an antigen rapid diagnostic test, the Kestrel^TM^ COVID-19 Ag Rapid Test, for professional use. The clinical performance of the test was compared with FDA-authorized RT-PCR by testing pre-confirmed residual nasopharyngeal swabs in viral transportation media (VTM) of COVID-19 infected patients. Tests were performed in two independent laboratories.

## 2. Materials and Methods

### 2.1. Ethics Statement

The prospective clinical study was conducted according to international standards of Good Clinical Practice at a Hat Yai tertiary hospital from August 2021 to September 2021. Ethical approval was granted by the Hat Yai Hospital Human Research Ethics Committee, Songkhla, Thailand (approval number: HYH EC 087-64-02).

### 2.2. Colloidal Gold Conjugation

Two types of colloidal gold nanoparticles were selected for sensitivity comparison: 40 nm colloidal gold nanoparticles (Kestrel Bio Sciences, Pathumthani, Thailand) and 150 nm gold Nanoshell (Nanocomposix, San Diego, CA, USA). The colloidal gold nanoparticles were prepared as follows. The 40 nm colloidal gold nanoparticles (1 OD) were adjusted to the appropriate pH for gold conjugation. MAb no.1 against SARS-CoV-2 NP (10 µg/mL) was conjugated with prepared colloidal gold nanoparticles (10 mL). The antibody-gold conjugates were precipitated by centrifugation and were suspended in borate buffer containing 1% bovine serum albumin (BSA) to adjust the OD of the conjugates to 10 at a wavelength of 450 nm. To prepare the conjugator pad, the conjugates were sprayed on a glass fiber membrane at a rate of 1 µL/mm and dried at 37 °C. 

The 150 nm gold Nanoshell was prepared following the protocol recommended by the manufacturer (Nanocomposix, San Diego, CA, USA). MAb no.1 against SARS-CoV-2 NP (20 µg) was conjugated with the 1 mL prepared gold Nanoshell. The antibody-gold conjugates were collected by centrifugation, and the desired OD for the final pellet was achieved in conjugate storage buffer. The conjugates were sprayed on a glass fiber membrane at a rate of 1 µL/mm and dried at 37 °C.

### 2.3. Assembly of Antigen Test Kit

MAb no.2 against SARS-CoV-2 NP (1.0 mg/mL) and Goat anti-mouse IgG (1.0 mg/mL; Lampire Biological laboratories, Pipersville, PA, USA) were dispensed and immobilized on a nitrocellulose membrane at the test line and the control line, respectively. The absorbance pad was untreated cotton paper (Grade 470, Whatman, Little Chalfont, UK), and the sample pad consisted of untreated glass fiber paper (Grade 8964, Ahlstrom-Munksjö, Helsinki, Finland). All pads were assembled so that they were partially overlapping to allow the flow of sample and buffer through the strip.

### 2.4. Limit of Detection (LOD)

The LOD of the developed test kit was determined by testing with the various concentrations of a recombinant SARS-CoV-2 nucleocapsid protein of wild-type SARS-CoV-2 (NP) (Leinco Technologies, Fenton, MO, USA) and Alpha-, Beta-, Gamma-, Delta-, Epsilon-, Kappa-, and Omicron variants of SARS-CoV-2 (ACRO Biosystems, Newark, DE, USA). The analytical sensitivity of the test kit was evaluated by comparing the concentration of recombinant SARS-CoV-2 NP antigen with the corresponding band intensity values of 0 ng/mL NP antigen. The intensities of the test lines were quantitated with 0 ng/mL NP by using the ImageJ Gel Analysis program. The lowest concentration of the recombinant NP antigen with positive values of quantitated band intensity was designated as the LOD of the Kestrel^TM^ COVID-19 Ag Rapid Test.

### 2.5. Cross-Reactivity and Interference Testing

Viral nucleocapsid antigens and bacterial whole-cell antigens were used for cross-reactivity and interference testing. The viral nucleocapsid protein antigens included SARS-CoV; MERS-CoV; common cold coronaviruses (HKU1, OC43, NL63, and 229E) (ACROBiosystems, Newark, DE, USA); Influenza A; Influenza B; and Parainfluenza virus 1–4 (MyBioSource, San Diego, CA, USA). The bacteria included *Haemophilus influenzae*, *Streptococcus pneumoniae*, *Streptococcus pyogenes*, *Candida albicans*, pooled human nasal wash—representative of normal respiratory microbial flora, *Mycobacterium tuberculosis*, *Staphylococcus aureus*, and *Staphylococcus epidermidis*. For cross-reactivity testing, the proteins and organisms were diluted to the final concentration in extraction buffer. One hundred microliters of the mixture was loaded into the sample well of the cassette. The result was read within 15 min. For interference testing, 3 × LOD concentration of a recombinant SARS-CoV-2 NP of the wild-type SARS-CoV-2 was added in the mixture of the above-mentioned viral and bacterial antigens. One hundred microliters of the mixture was loaded into the sample well of the cassette. The result was read within 15 min. The tests were performed in triplicate for each sample.

### 2.6. Clinical Specimens

Clinical evaluation was performed in the immunological and virological laboratory in the Department of Medical Technology and Clinical Pathology of Hat Yai Hospital, a tertiary hospital in Songkhla, Thailand. The laboratory has passed the external quality control of RT-PCR tests by the Department of Medical Science (DMSC), Ministry of Public Health, and has been authorized as a COVID-19 diagnostic laboratory [15]. Nasopharyngeal swabs were collected from suspected COVID-19 infection from August to September 2021 at Hat Yai Hospital and Ramathibodi hospital. Participant inclusion criteria consisted of being over 18 years of age, visiting a doctor, with or without symptoms, and/or presenting suspected COVID-19 infection within 10 days of symptom onset. No requirement specific to gender was set. Participant exclusion criteria consisted of the inability to tolerate sample collection and any process where leaky tubes or sample contamination was found. Nasopharyngeal specimens were collected by trained healthcare providers following the Center for Disease Control and Prevention (CDC)’s interim guidelines for the collecting and handling of clinical specimens for COVID-19 testing [16]. The specimens were transported in 2 mL of viral transport media (VTM) at 2–8 °C to the laboratory and were processed immediately after arrival. All specimens were processed in Biosafety Level-2 Enhanced or BSL-2+ facility, using full personal protective equipment, in a laboratory qualified for COVID-19 detection.

### 2.7. Viral RNA Extraction

SARS-CoV-2 RNA was extracted from 200 µL of nasopharyngeal specimens using EXM3000 automated nucleic acid isolation system, via magnetic bead method (Zybio Inc., Chongqing, China), following the manufacturer’s instructions. Extracted viral RNA samples were used as a template for real-time RT-PCR assay using the FDA-authorized Novel Coronavirus (2019-nCoV) nucleic acid diagnostic kit PCR-Fluorescence Probing (Sansure Biotech, Hunan, China). Ten microliters of extracted RNA and real-time RT-PCR reagents were prepared in a NATCH CS automated system (Sansure Biotech, Hunan, China).

### 2.8. Real-Time PCR Detection of SARS-CoV-2 RNA Detection

Target genes for SARS-CoV-2 RNA detection were ORF lab and N gene. The internal standard gene fragments were Rnase P. Real-time RT-PCR assay using the Novel Coronavirus (2019-nCoV) nucleic acid diagnostic kit PCR-Fluorescence Probing (Sansure Biotech, Hunan, China) was performed according to the manufacturer’s instructions.

Briefly, for one test, 26 µL of PCR mix and 4 µL of the enzyme were thoroughly mixed, after which 30 µL PCR-Mastermix was added to 20 µL of extracted RNA sample. The PCR conditions consisted of 1 cycle of 30 min at 50 °C for reverse transcription, 1 cycle of 1 min at 95 °C for cDNA pre-denaturation, followed by 45 cycles of 15 s at 95 °C, and 30 s at 60 °C. The SLAN^®^-96P Real-Time PCR System (Sansure Biotech, Hunan, China) was used for PCR amplification. A positive result for SARS-CoV-2 was defined as the detection of a typical S-shape amplification curve produced by the internal control Rnase P gene, and ORF lab and/or N gene detected at Ct < 40.

### 2.9. Clinical Evaluation Study of the Rapid Antigen Test

Nasopharyngeal specimens were collected from 319 samples, 159 samples of suspected COVID-19 infection from Hat Yai Hospital, Songkhla province, and 160 samples from Ramathibodi Hospital, Bangkok, Thailand. The clinical evaluation results of the test kit by Hat Yai Hospital and Ramathibodi Hospital are shown in Table 1 and Table 2, respectively. Fifty-nine positive and 100 negative nasopharyngeal specimens confirmed by the real-time RT-PCR assay PCR-Fluorescence Probing (Sansure Biotech, Hunan, China) were selected and analyzed within 24 h with the Kestrel^TM^ COVID-19 Ag Rapid Test. The result was compared with the real-time RT-PCR assay simultaneously. All specimens were processed in Biosafety Level-2 Enhanced (BSL-2+) facilities. Moreover, nasopharyngeal specimens of 160 cases (60 positive and 100 negative cases) were used for evaluation of the antigen test kit by Ramathibodi Hospital, Bangkok, Thailand.

**Figure 1:**
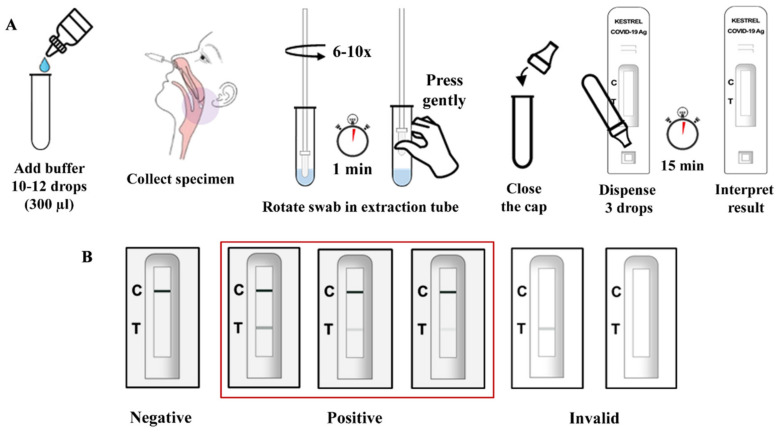
Diagram of assay procedure (**A**) and result interpretation (**B**) of Kestrel^TM^ COVID-19 Ag Rapid Test.

A nasopharyngeal swab was collected from the patient and transported to the laboratory in a VTM tube. Then, the nasopharyngeal swab was transferred into the extraction tube containing extraction buffer, was mixed thoroughly in a test tube, and stood for 1 min. One hundred microliters of the mixture were loaded into the sample well of the cassette, and the test result was read within 15 min. The appearance of two-colored lines, control (C) and test (T), was considered a positive result and indicated that SARS-CoV-2 antigens were present in the specimen (Figure 1). Clinical performance of the Kestrel^TM^ COVID-19 Ag Rapid Test was assessed by two authorized COVID-19 diagnostic laboratories located in Hat Yai Hospital, Songkhla province, and Ramathibodi Hospital, Bangkok, respectively.

### 2.10. Statistical Analysis

Sensitivity, specificity, positive predictive value (PPV), negative predictive value (NPV), and 95% confidence interval (95% CI) were calculated as follows:

Diagnostic sensitivity (%) = true positive/(true positive + false negative) × 100

Diagnostic specificity (%) = true negative/(false positive + true negative) × 100

Positive predictive value (%) = true positive/(true positive + false positive) × 100

Negative predictive value (%) = true negative/(false negative + true negative) × 100

According to the announcement of the Food and Drug Administration, Ministry of Public Health, Thailand, the criteria for the evaluation of the clinical sensitivity and specificity of the SARS-CoV-2 antigen detection test kit are as follows: (1) diagnostic sensitivity: ≥90%, *n* ≥ 50; (2) diagnostic specificity: ≥98%, *n* ≥ 100; and (3) non-specificity: ≤10%, *n* ≥ 20 (*n* = number of samples tested).

## 3. Results

### 3.1. Selection of Gold Nanoparticles

**Figure 2:**
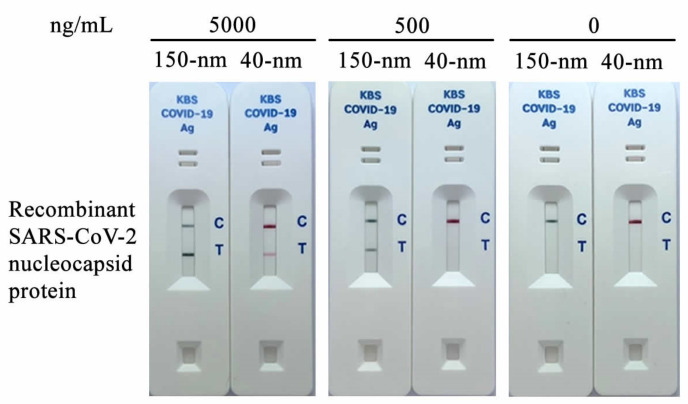
Gold nanoparticle selection. The 150 nm gold Nanoshell (dark green-colored) and 40 nm colloidal gold (red-colored) were tested with 5000, 500, and 0 ng/mL of recombinant nucleocapsid protein.

The quality of gold nanoparticles is one of the crucial factors in determining the sensitivity of lateral flow detection. Commonly used 40 nm colloidal gold (red-colored) and 150 nm gold Nanoshell (dark green-colored) were compared in order to select for high-sensitivity SARS-CoV-2 detection. The antibody-gold Nanoshell conjugates could detect recombinant SARS-CoV-2 NP antigen clearly at 500 µg/mL, in contrast to the 40 nm colloidal gold conjugates (Figure 2). Therefore, the gold Nanoshell has been further used for developing the test kit due to its higher detection sensitivity.

### 3.2. Limit of Detection (LOD) and Variant Detection of the Kestrel^TM^ COVID-19 Ag Rapid Test Kit

**Figure 3:**
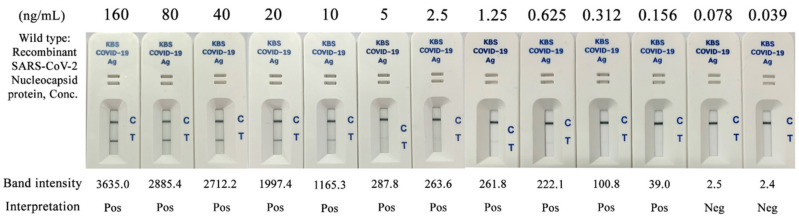
Limit of detection of Kestrel^TM^ COVID-19 Ag Rapid Test using recombinant SARS-CoV-2 nucleocapsid protein.

The recombinant SARS-CoV-2 nucleocapsid protein (NP) was diluted to concentrations ranging from 160 ng/mL to 0.039 ng/mL. The lowest concentration detected by the Kestrel^TM^ COVID-19 Ag Rapid Test was 0.156 ng/mL, with a band intensity of 39.0 (Figure 3). Therefore, the LOD of the Kestrel^TM^ COVID-19 Ag Rapid Test was 0.156 ng/mL, or approximately 0.2 ng/mL for recombinant wild-type SARS-CoV-2 nucleocapsid protein.

**Figure 4:**
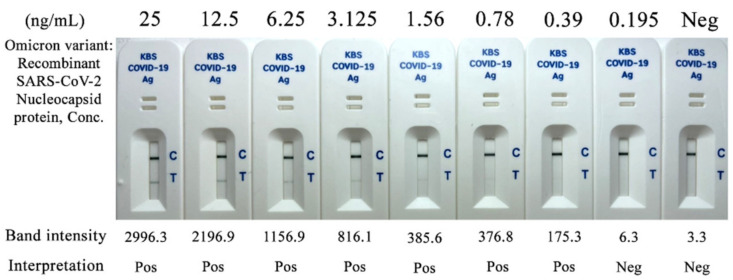
Detection of Kestrel^TM^ COVID-19 Ag Rapid Test using recombinant SARS-CoV-2 nucleocapsid protein of Omicron-variant of SARS-CoV-2.

The Kestrel^TM^ COVID-19 Ag Rapid Test kit successfully detected the recombinant SARS-CoV-2 nucleocapsid protein (NP) of wild-type and the tested variants as positive results. The LOD of the test was 0.156 ng/mL for recombinant SARS-CoV-2 nucleocapsid protein of Alpha-, Beta-, Gamma-, Delta-, Epsilon-, Kappa-variant, and 0.39 ng/mL for the Omicron-variant, respectively. The LOD of the Omicron variant, the new variant of concern, is shown in Figure 4.

### 3.3. Cross-Reactivity and Interference

Viral nucleocapsid protein antigens of MERS-CoV, common cold coronaviruses HKU1, OC43, NL63, and 229E; Influenza A; Influenza B; and Parainfluenza virus 1–4; and the whole bacterial cells *Haemophilus influenzae*, *Streptococcus pneumoniae*, *Streptococcus pyogenes*, *Candida albicans*, pooled human nasal wash, *Mycobacterium tuberculosis*, *Staphylococcus aureus*, and *Staphylococcus epidermidis* all have been tested but did not produce any positive results of cross-reactivity; hence, no detection was shown by the test kit (Supplementary Table S1). Likewise, all proteins of the organisms listed above did not interfere with a positive result of SARS-CoV-2 when tested in the same reaction. However, the test kit showed a positive result for SARS-CoV. Therefore, the test did not differentiate between SARS-CoV and SARS-CoV-2. 

### 3.4. Clinical Evaluation of the Kestrel^TM^ COVID-19 Ag Rapid Test Kit

#### 3.4.1. Real-Time RT-PCR

All clinical samples used for antigen test kit evaluation were confirmed by real-time RT-PCR for the detection of SARS-CoV-2. The average cycle threshold (Ct) values for SARS-CoV-2 positive samples were 20.55 ± 3.711 (min 12.28, max 26.67) for the *ORF1AB* gene and 17.59 ± 3.41 (min 10.77, max 23.88) for the *N* gene (Supplementary Table S2). The samples containing Ct values higher than 40 for both target genes were considered negative for real-time RT-PCR.

#### 3.4.2. Kestrel^TM^ COVID-19 Antigen Test Kit

The SARS-CoV-2 antigen detection performance of the Kestrel^TM^ COVID-19 Ag Rapid Test was evaluated using real-time RT-PCR-confirmed nasopharyngeal samples. The results were interpreted within 30 min of detection upon a clear appearance of the control line. Of the 59 RT-PCR positive samples from Hat Yai Hospital, 57 samples were detected positive for the SARS-CoV-2 antigen by the test kit, while two were false-negatives (Table 1 and Supplementary Table S2). Of the 60 RT-PCR positive samples from Ramathibodi Hospital, 59 samples were detected positive for the SARS-CoV-2 antigen by the test kit, while one was a false-negative (Table 2). All RT-PCR negative samples were also negative for SARS-CoV-2 antigen by the test kit. The overall detection sensitivity and specificity of Kestrel^TM^ COVID-19 Ag Rapid Test from two independent laboratories were 96.49% and 98.33% of sensitivity, and 100% and 100% of specificity, respectively, as shown in Table 3. The overall accuracy of the test kit was 99.01% and 99.44%, as determined by Hat Yai Hospital and Ramathibodi Hospital, respectively.

## 4. Discussion

Rapid tests are widely used for the early detection and screening of COVID-19 infection prior to the RT-PCR confirmatory test [17,18]. Fast turnaround time and ease of use are important features of antigen tests, which enable large-scale rapid screening of suspected infections; however, the rapid test must be integrated into proper strategies for transmission control [19]. Consequently, such tests need to be of high quality to ensure effective control of the COVID-19 outbreak. While rapid test kits of varying quality have been available worldwide, their false-positive results and missed positive cases diminish their potential effectiveness for fieldwork [20,21]. Therefore, the development of a reliable, high-quality antigen test kit was needed, forming the purpose of this study.

To detect positive cases effectively, the COVID-19 antigen test kit needs to be highly sensitive. A potential sensitivity issue of test kits that target the COVID-19 spike protein has become increasingly apparent through the emergence of VOCs with many spike protein substitutions, including Omicron [22]. Therefore, our test kit was designed to target the nucleocapsid protein instead, which has a low mutation rate, is highly immunogenic, and is abundantly expressed during SARS infections, potentially resulting in a higher sensitivity of detection [23,24].

The nucleocapsid protein of Alpha, Beta, Gamma, Delta, Epsilon, Kappa, and Omicron variants of SARS-CoV-2 were detected by our newly developed rapid test, demonstrating the ability of the Kestrel^TM^ COVID-19 Ag Rapid Test kit to effectively detect infection by both SARS-CoV-2 wild-type and its variants. 

Besides the target protein, the gold nanoparticle selection also impacts the limit of detection of the test. Highly stable monoclonal antibody conjugates using carboxyl Gold Nanoshell provide a 20-fold increase in sensitivity over the 40 nm gold commonly used in lateral flow assay [25]. The LOD of the Kestrel^TM^ COVID-19 Ag Rapid Test kit was determined to be approximately 0.2 ng/mL for recombinant SARS-CoV-2 nucleocapsid protein.

One key aspect of the successful development of lateral flow assays is a sensitive and stable conjugate. To obtain a stable conjugate, the conjugation process needs to be optimized. General lateral flow assays prefer passive conjugation because it is a quick and easy method. However, to maximize sensitivity, passive adsorption results in high antibody loading on the surface of gold nanoparticles. In contrast, covalent conjugation used in this study requires fewer antibodies and the stability of antibody-conjugated gold nanoparticles increases. As a result, the sensitivity of our proposed method was markedly high (96.49% and 98.33%) compared to that of other commercially available diagnostic kits using the passive adsorption method (90.00%; 95% CI: 73.47% to 97.89%) [12].

The sensitivity and specificity of our evaluated test kit reached and exceeded the values recommended by Thai FDA guidelines for the evaluation of COVID-19 antigen test kits, which recommends a minimum of 90% diagnostic sensitivity and 98% diagnostic specificity. Clinical evaluation of our test kit by two independent laboratories showed closely related results of 96.49% and 98.33% of sensitivity, 100% and 100% of specificity, and 99.01% and 99.44% of accuracy determined by Hat Yai Hospital and Ramathibodi Hospital, respectively.

Several factors impacting the likelihood of false results during field use have been reported, including inadequate specimen collection, period of detection, logistical and storage conditions of the test, and laboratory errors [26,27]. Well-trained health professionals are essential to perform the proper technique to reach the target site of the nasopharynx [28]. The optimal testing window of nasopharyngeal swab collection is three days after symptom onset, and even so, a false-negative result could be as high as 21% [29]. The recommended temperature for the storage of antigen test kits ranges between 2 °C to 30 °C. Exposure to 37 °C, even for a short time, reduces the sensitivity of the SARS-CoV-2 antigen test. Thus, the storage and operation of antigen test kits at recommended conditions is essential [30]. Error in test performance, such as improper sample handling, or the contamination of test kits or clinical specimens, could also produce false test results [31].

No diagnostic test can be performed properly without appropriate care by the user. Following the manufacturer’s instructions ensures accurate results, but it is important to keep in mind that instructions by different manufacturers are not interchangeable [14]. While the performance of antigen tests is evaluated by qualified laboratories under carefully controlled conditions, their sensitivity and specificity could be slightly different on test operation sites. Known positive and negative samples should be tested with the kit when operating at a new location or when switching to a new lot of test kit. Therefore, internal quality control is necessary for their optimal operation on testing sites.

## 5. Conclusions

The evaluation results successfully demonstrate the effectiveness of the Kestrel^TM^ COVID-19 Ag Rapid Test kit with high sensitivity (96.49%; 95%CI, 88.29% to 99.59%, and 98.33%; 95% CI, 91.06–99.96%) and specificity (100%, 95% CI, 96.38–100%) comparable to real-time RT-PCR evaluated by two independent laboratories. Due to its rapid deployment, ease of use, and wide range of SARS-CoV-2 variant detection, this antigen test may have potential use for COVID-19 screening, surveillance, and infection control.

## Figures and Tables

**Table 1:** Clinical evaluation results of Kestrel^TM^ COVID-19 Ag Rapid Test by Hat Yai Hospital.

Kestrel^TM^ COVID-19 Antigen Test	RT-PCR (Sansure^®^ Novel Coronavirus (2019-nCoV) Nucleic Acid Diagnostic Kit		Total
	Positive	Negative	
Positive	57	0	57
Negative	2	100	102
Total	59	100	159

**Table 2:** Clinical evaluation results of Kestrel^TM^ COVID-19 Ag Rapid Test by Ramathibodi Hospital.

Kestrel^TM^ COVID-19 Antigen Test	RT-PCR (Sansure^®^ Novel Coronavirus (2019-nCoV) Nucleic Acid Diagnostic Kit		Total
	Positive	Negative	
Positive	59	0	59
Negative	1	100	101
Total	60	100	160

**Table 3:** Clinical evaluation results of the Kestrel^TM^ COVID-19 Ag Rapid Test from different laboratories.

Clinical Evaluation	Hat Yai Hospital	Ramathibodi Hospital	Accepted Criteria by Thai FDA
%Sensitivity (95%CI)	96.49% (87.89–99.57%)	98.33% (91.06–99.96%)	≥90%
Specificity (95%CI)	100% (97.49–100%)	100% (96.38–100%)	≥98%
Accuracy (95%CI)	99.01% (96.47–99.88%)	99.44% (NR) *	-

* NR = Not reported.

## Data Availability

Data supporting the reported results may be provided upon reasonable request to the corresponding author.

## Supplementary Materials

The following supporting information can be downloaded at: www.mdpi.com/article/10.3390/diagnostics12020381/s1, Table S1: Cross-reactivity and interference testing for the Kestrel^TM^ COVID-19 Ag Rapid Test Kit; Table S2: The cycle threshold (Ct) values for SARS-CoV-2 positive samples for *ORF1AB* gene, and *N* gene by real-time RT-PCR compared with Kestrel^TM^ COVID-19 Ag Rapid Test kit.
