# Supplementary material for: Development, Analytical, and Clinical Evaluation of Rapid Immunochromatographic Antigen Test for SARS-CoV-2 Variants Detection
Source: Diagnostics (Basel). 2022 Feb 2;12(2):381. doi: 10.3390/diagnostics12020381 (PMC8871278; doi:10.3390/diagnostics12020381)
Supplement: Supplementary file 1 [file diagnostics-12-00381-s001.zip › Supplementary Table S2_diagnostics-1555854_edited.pdf]

Supplementary Table S2 Clinical data and cycle threshold (Ct) values for SARS-CoV-2 positive samples for *ORF1AB* gene and *N* gene by real-time RT-PCR compared with the Kestrel™ COVID-19 Ag Rapid Test Kit.

Sample no.	Specimen ID	Gender	Age	Date of collection	Date of Ag test kit Process	Kestrel Ag rapid test	Date of RT-PCR process	RT-PCR Ct value (Sansure®)		Internal control (IC)	RT-PCR interpretation	Patient symptoms
								ORF1AB	N gene			
1	SARS-CoV-2-Pos-1	F	47	3/08/2021	3/08/2021	Positive	2/08/2021	17.88	15.18	27.09	Positive	Fever, cough, congestion, headache, shortness of breath
2	SARS-CoV-2-Pos-2	F	27	3/08/2021	3/08/2021	Positive	2/08/2021	22.71	18.06	28.56	Positive	Asymptomatic
3	SARS-CoV-2-Pos-3	F	43	3/08/2021	3/08/2021	Positive	2/08/2021	22.47	19.47	25.56	Positive	Asymptomatic
4	SARS-CoV-2-Pos-4	M	31	3/08/2021	3/08/2021	Positive	2/08/2021	21.79	18.82	25.98	Positive	N/A*
5	SARS-CoV-2-Pos-5	M	26	3/08/2021	3/08/2021	Positive	2/08/2021	24.56	20.82	25.1	Positive	N/A
6	SARS-CoV-2-Pos-6	F	26	3/08/2021	3/08/2021	Positive	3/08/2021	14.91	11.1	24.22	Positive	N/A
7	SARS-CoV-2-Pos-7	M	32	3/08/2021	3/08/2021	Positive	3/08/2021	16.82	13.5	28.78	Positive	N/A
8	SARS-CoV-2-Pos-8	F	35	3/08/2021	3/08/2021	Positive	3/08/2021	18.04	15.17	24.72	Positive	N/A
9	SARS-CoV-2-Pos-9	F	74	3/08/2021	3/08/2021	Positive	3/08/2021	18.15	14.84	24.28	Positive	N/A
10	SARS-CoV-2-Pos-10	F	30	3/08/2021	3/08/2021	Positive	3/08/2021	22.77	19	26.42	Positive	N/A
11	SARS-CoV-2-Pos-11	F	33	3/08/2021	3/08/2021	Positive	3/08/2021	19.23	16.06	26.19	Positive	N/A
12	SARS-CoV-2-Pos-12	F	27	3/08/2021	3/08/2021	Positive	3/08/2021	26.4	23.73	25.04	Positive	N/A
13	SARS-CoV-2-Pos-13	M	72	4/08/2021	4/08/2021	Positive	4/08/2021	26.31	23.44	28.28	Positive	Fever, headache, cough
14	SARS-CoV-2-Pos-14	F	63	5/08/2021	5/08/2021	Positive	5/08/2021	13.8	11.59	26.77	Positive	Asymptomatic
15	SARS-CoV-2-Pos-15	M	29	5/08/2021	5/08/2021	Positive	5/08/2021	17.55	13.42	28.25	Positive	Asymptomatic
16	SARS-CoV-2-Pos-16	F	30	5/08/2021	5/08/2021	Positive	5/08/2021	25.11	22.42	26.27	Positive	Asymptomatic
17	SARS-CoV-2-Pos-17	F	39	5/08/2021	5/08/2021	Positive	5/08/2021	17.73	15.16	28.01	Positive	Asymptomatic
18	SARS-CoV-2-Pos-18	M	27	5/08/2021	5/08/2021	Negative	5/08/2021	26.67	23.88	26.97	Positive	N/A
19	SARS-CoV-2-Pos-19	F	45	7/08/2021	7/08/2021	Positive	7/08/2021	25.22	21.99	28.21	Positive	N/A
20	SARS-CoV-2-Pos-20	M	64	7/08/2021	7/08/2021	Positive	7/08/2021	20.53	17.68	27.25	Positive	N/A
21	SARS-CoV-2-Pos-21	F	31	7/08/2021	7/08/2021	Positive	7/08/2021	21.29	17.2	26.71	Positive	N/A
22	SARS-CoV-2-Pos-22	F	24	13/08/2021	13/08/2021	Positive	13/08/2021	21.4	17.72	27.99	Positive	N/A
23	SARS-CoV-2-Pos-23	F	33	13/08/2021	13/08/2021	Positive	13/08/2021	22.57	19.58	27.39	Positive	Asymptomatic
24	SARS-CoV-2-Pos-24	M	18	13/08/2021	13/08/2021	Positive	13/08/2021	15.66	13.14	27.69	Positive	N/A
25	SARS-CoV-2-Pos-25	M	26	13/08/2021	13/08/2021	Positive	13/08/2021	24.86	21.87	31.3	Positive	Asymptomatic
26	SARS-CoV-2-Pos-26	F	57	13/08/2021	13/08/2021	Positive	13/08/2021	16.15	13.33	28.45	Positive	Asymptomatic
27	SARS-CoV-2-Pos-27	F	44	13/08/2021	13/08/2021	Positive	13/08/2021	23.67	20.69	26.72	Positive	N/A
28	SARS-CoV-2-Pos-28	M	71	15/08/2021	15/08/2021	Positive	15/08/2021	21.69	18.69	27.07	Positive	Cough, congestion, muscle aches, fatigue
29	SARS-CoV-2-Pos-29	F	54	15/08/2021	15/08/2021	Positive	15/08/2021	20.4	17	29.22	Positive	N/A
30	SARS-CoV-2-Pos-30	F	74	17/08/2021	17/08/2021	Positive	17/08/2021	23.22	19.57	29.86	Positive	Cough, congestion, headache, shortness of breath, sore throat
31	SARS-CoV-2-Pos-31	F	18	17/08/2021	17/08/2021	Negative	17/08/2021	26.17	23.34	26.57	Positive	Asymptomatic
32	SARS-CoV-2-Pos-32	F	23	17/08/2021	17/08/2021	Positive	17/08/2021	24.83	21.79	26.63	Positive	Asymptomatic
33	SARS-CoV-2-Pos-33	F	30	17/08/2021	17/08/2021	Positive	17/08/2021	19.33	16.66	28.58	Positive	N/A

34	SARS-CoV-2-Pos-34	F	20	17/08/2021	17/08/2021	Positive	17/08/2021	23.88	21.27	26.38	Positive	N/A
35	SARS-CoV-2-Pos-35	M	18	17/08/2021	17/08/2021	Positive	17/08/2021	12.28	18.1	27.07	Positive	Asymptomatic
36	SARS-CoV-2-Pos-36	M	51	17/08/2021	17/08/2021	Positive	17/08/2021	19.29	15.78	31.86	Positive	Asymptomatic
37	SARS-CoV-2-Pos-37	F	48	17/08/2021	17/08/2021	Positive	17/08/2021	18.91	15.03	26.91	Positive	Asymptomatic
38	SARS-CoV-2-Pos-38	F	37	20/08/2021	20/08/2021	Positive	20/08/2021	24.39	20.81	28.49	Positive	Fever, cough, congestion, headache
39	SARS-CoV-2-Pos-39	F	29	20/08/2021	20/08/2021	Positive	20/08/2021	21.15	17.6	28.75	Positive	Muscle ache, headache
40	SARS-CoV-2-Pos-40	M	33	20/08/2021	20/08/2021	Positive	20/08/2021	19.63	16.58	27.98	Positive	Fever, cough, congestion
41	SARS-CoV-2-Pos-41	F	32	20/08/2021	20/08/2021	Positive	20/08/2021	23.44	20.54	27.53	Positive	Fever, cough, congestion
42	SARS-CoV-2-Pos-42	M	23	20/08/2021	20/08/2021	Positive	20/08/2021	17.02	14.02	27.87	Positive	Asymptomatic
43	SARS-CoV-2-Pos-43	M	40	20/08/2021	20/08/2021	Positive	20/08/2021	26.65	21.94	26.41	Positive	Asymptomatic
44	SARS-CoV-2-Pos-44	M	43	20/08/2021	20/08/2021	Positive	20/08/2021	18.64	15.46	28.86	Positive	Asymptomatic
45	SARS-CoV-2-Pos-45	F	26	25/08/2021	25/08/2021	Positive	25/08/2021	22.84	19.86	25.56	Positive	N/A
46	SARS-CoV-2-Pos-46	F	18	25/08/2021	25/08/2021	Positive	25/08/2021	23.1	20.21	26.21	Positive	N/A
47	SARS-CoV-2-Pos-47	F	32	25/08/2021	25/08/2021	Positive	25/08/2021	14.16	10.77	24.22	Positive	N/A
48	SARS-CoV-2-Pos-48	F	52	26/08/2021	26/08/2021	Positive	26/08/2021	21.031	15.46	27.27	Positive	Fever, cough, congestion
49	SARS-CoV-2-Pos-49	F	23	29/08/2021	29/08/2021	Positive	29/08/2021	13.14	19.73	25.69	Positive	Fever, cough, sore throat, headache, congestion
50	SARS-CoV-2-Pos-50	F	45	29/08/2021	29/08/2021	Positive	29/08/2021	18.31	13.69	27.82	Positive	Fever, cough, congestion
51	SARS-CoV-2-Pos-51	M	75	2/09/2021	2/09/2021	Positive	2/09/2021	23.87	19.74	24.85	Positive	Cough, sore throat, headache, congestion, fatigue
52	SARS-CoV-2-Pos-52	M	24	2/09/2021	2/09/2021	Positive	2/09/2021	19.57	16.61	27.27	Positive	Congestion, loss of taste/smell
53	SARS-CoV-2-Pos-53	F	25	2/09/2021	2/09/2021	Positive	2/09/2021	21.85	19.01	26.92	Positive	Fever, cough, congestion
54	SARS-CoV-2-Pos-54	M	27	7/09/2021	7/09/2021	Positive	7/09/2021	18.95	14.65	26.65	Positive	Asymptomatic
55	SARS-CoV-2-Pos-55	M	19	7/09/2021	7/09/2021	Positive	7/09/2021	15.58	13.24	25.03	Positive	Asymptomatic
56	SARS-CoV-2-Pos-56	F	29	7/09/2021	7/09/2021	Positive	7/09/2021	15.95	13.24	25.22	Positive	N/A
57	SARS-CoV-2-Pos-57	M	28	7/09/2021	7/09/2021	Positive	7/09/2021	22.27	18.83	26.8	Positive	Fever, headache, cough
58	SARS-CoV-2-Pos-58	F	44	7/09/2021	7/09/2021	Positive	7/09/2021	19.3	16.16	29.39	Positive	Headache, sore throat
59	SARS-CoV-2-Pos-59	F	52	7/09/2021	7/09/2021	Positive	7/09/2021	17.13	13.83	29.02	Positive	N/A

*N/A = not available
