# Supplementary material for: Development, Analytical, and Clinical Evaluation of Rapid Immunochromatographic Antigen Test for SARS-CoV-2 Variants Detection
Source: Diagnostics (Basel). 2022 Feb 2;12(2):381. doi: 10.3390/diagnostics12020381 (PMC8871278; doi:10.3390/diagnostics12020381)
Supplement: Supplementary file 1 [file diagnostics-12-00381-s001.zip › Supplementary TableS1_diagnostics-1555854_edited.pdf]

Supplementary Table S1 Cross reactivity and interference testing for the Kestrel™ COVID-19 Ag Rapid Test Kit.

Potential cross-reactant	Concentration	Cross-reactivity (Yes/No)	Interference (Yes/No)
Other viral nucleocapsid antigen			
SARS-coronavirus NP antigen	10 µg/mL	Yes (3/3 positive)	No (3/3 positive)
MERS-coronavirus NP antigen	10 µg/mL	No (3/3 negative)	No (3/3 positive)
Human coronavirus 229E NP antigen	10 µg/mL	No (3/3 negative)	No (3/3 positive)
Human coronavirus OC43 NP antigen	10 µg/mL	No (3/3 negative)	No (3/3 positive)
Human coronavirus NL63 NP antigen	10 µg/mL	No (3/3 negative)	No (3/3 positive)
Parainfluenza virus 1 NP antigen	180 µg/mL	No (3/3 negative)	No (3/3 positive)
Parainfluenza virus 2 NP antigen	180 µg/mL	No (3/3 negative)	No (3/3 positive)
Parainfluenza virus 3 NP antigen	180 µg/mL	No (3/3 negative)	No (3/3 positive)
Parainfluenza virus 4 NP antigen	180 µg/mL	No (3/3 negative)	No (3/3 positive)
Influenza A NP antigen	180 µg/mL	No (3/3 negative)	No (3/3 positive)
Influenza B NP antigen	180 µg/mL	No (3/3 negative)	No (3/3 positive)
Other bacteria and yeast (whole cell protein)			
Haemophilus influenzae	1.5 x 10^8 CFU/mL	No (3/3 negative)	No (3/3 positive)
Streptococcus pneumoniae	1.5 x 10^8 CFU/mL	No (3/3 negative)	No (3/3 positive)
Streptococcus pyogenes	1.5 x 10^8 CFU/mL	No (3/3 negative)	No (3/3 positive)
Candida albicans	10^6 CFU/mL	No (3/3 negative)	No (3/3 positive)
Pooled human nasal wash – representative of normal respiratory microbial flora	100%	No (3/3 negative)	No (3/3 positive)
Mycobacterium tuberculosis	>2×10^3 CFU/mL	No (3/3 negative)	No (3/3 positive)
Staphylococcus aureus	1.5 x 10^8 CFU/mL	No (3/3 negative)	No (3/3 positive)
Staphylococcus epidermidis	1.5 x 10^8 CFU/mL	No (3/3 negative)	No (3/3 positive)
